# Supplementary material for: Enhancing Silicon Solar Cell Performance Using a Thin-Film-like Aluminum Nanoparticle Surface Layer
Source: Nanomaterials (Basel). 2024 Feb 6;14(4):324. doi: 10.3390/nano14040324 (PMC10891793; doi:10.3390/nano14040324)
Supplement: Supplementary file 1 [file nanomaterials-14-00324-s001.zip › nanomaterials-2834221-supplementary.pdf]

M. D. Fjell[1], J. B. Lothe[1], N. J. Halas[2], M. H. Rosnes[3], B. Holst[1], M. M. Greve[1,*]

Enhancing Silicon Solar Cell Performance Using a Thin Film-Like Aluminum Nanoparticle Surface Layer

Supplementary Material

S 1. Nanoparticle Interaction with Light

Figure S1 The anti-reflection coating (ARC) thickness is 75 nm, and the surface coverage is 12.6% (array period 350 nm). The general trend for all oxide thicknesses is that there is an increase in light transmission below ~500 nm and above ~750 nm. In the wavelength region where the ARC is designed to be most effective, around 600 nm, there is a reduction in light transmission. The features that start at around 350 nm for all the oxide thicknesses are Wood-Rayleigh anomalies. Wood-Rayleigh anomalies are an in-plane diffraction component that can be seen as dips and peaks in the spectrum [46]. The anomalies depend on the array period h and the permittivity of the surrounding medium ε_m. For normal incident light on a square array of nanoparticles, the spectral position of the anomalies is given by

$$\lambda_{s,q} = h\sqrt{\frac{\varepsilon_m}{s^2 + q^2}}, \tag{S1}$$

where s and q are integers representing the constructive phase difference order in x and y directions. Hence, the wavelength at which the anomalies occur decreases with increasing phase difference order.

Figure S2 shows the angular scattering distribution of the electric field intensity $|\mathrm{E}|^2$ for an Al nanoparticle with (a) a 14 nm and (b) a 42 nm thick oxide layer on top of a solar cell at wavelength 613 nm, where the ARC is most efficient. These are the oxide layer thicknesses yielding the smallest and largest increase in transmission for a 140 nm nanoparticle (see Fig. 4). Clearly, the side lobes in the forward direction are larger for a 14 nm oxide layer than for a 42 nm oxide layer, consequently reducing the efficiency of the ARC most in the former case (see discussion in Section 4.2). In addition, the maximum forward to maximum backward ratio is 3.1 for a 14 nm oxide layer and 5.2 for a 42 nm oxide layer. Hence, a larger portion of the incoming light is reflected for the nanoparticles with a 14 nm oxide layer than for the ones with a 42 nm oxide layer, further explaining the difference in total transmission for the two cases.

Error! Reference source not found. shows the transmission of light into crystalline silicon (c-Si) as a function of wavelength for a bare solar cell and for a solar cell with 140 nm Al nanoparticles with different oxide thicknesses. The anti-reflection coating (ARC) thickness is 75 nm, and the surface coverage is 12.6% (array period 350 nm). The general trend for all oxide thicknesses is that there is an increase in light transmission below ~500 nm and above ~750 nm. In the wavelength region where the ARC is designed to be most effective, around 600 nm, there is a reduction in light transmission. The features that start at around 350 nm for all the oxide thicknesses are Wood-Rayleigh anomalies. Wood-Rayleigh anomalies are an in-plane diffraction component that can be seen as dips and peaks in

[1] Department of Physics and Technology, University of Bergen, P.O.Box 7803, 5020 Bergen, Norway
[2] Department of Electrical and Computer Engineering, Rice University, Houston, Texas 77005, United States
[3] Department of Chemistry, University of Bergen, P.O. Box 7803, 5020 Bergen, Norway
[*] e-mail: martin.greve@uib.no

the spectrum [46]. The anomalies depend on the array period h and the permittivity of the surrounding medium ε_m. For normal incident light on a square array of nanoparticles, the spectral position of the anomalies is given by

$$\lambda_{s,q} = h\sqrt{\frac{\varepsilon_m}{s^2 + q^2}}, \tag{S1}$$

where s and q are integers representing the constructive phase difference order in x and y directions. Hence, the wavelength at which the anomalies occur decreases with increasing phase difference order.

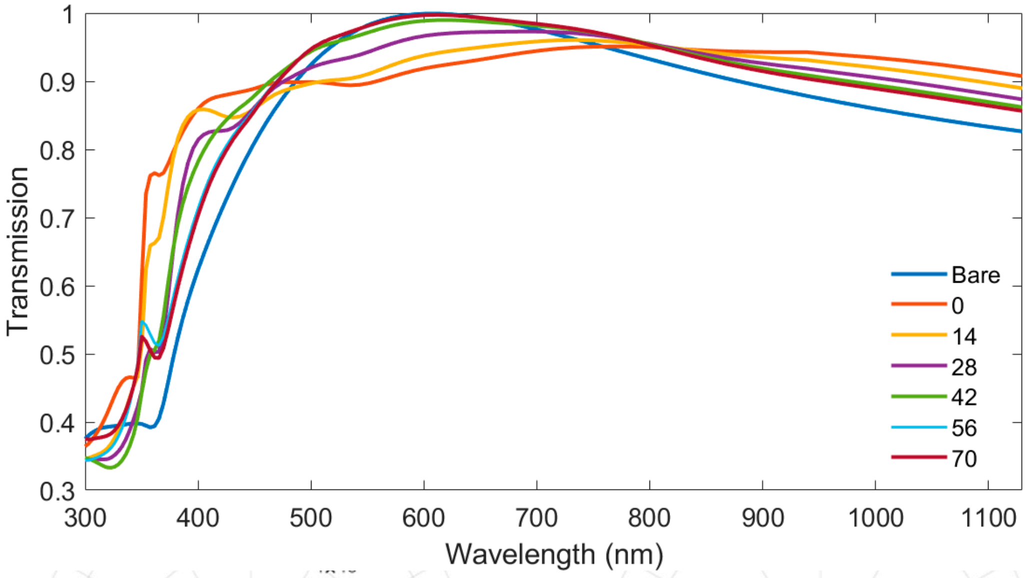

Figure S1: The transmission of light into crystalline silicon (c-Si) as a function of wavelength for a bare solar cell and for a solar cell with Al nanoparticles with varying oxide thickness. The nanoparticles have a diameter of 140 nm and a 21.0% surface coverage, and the anti-reflective coating (ARC) thickness is 75 nm.

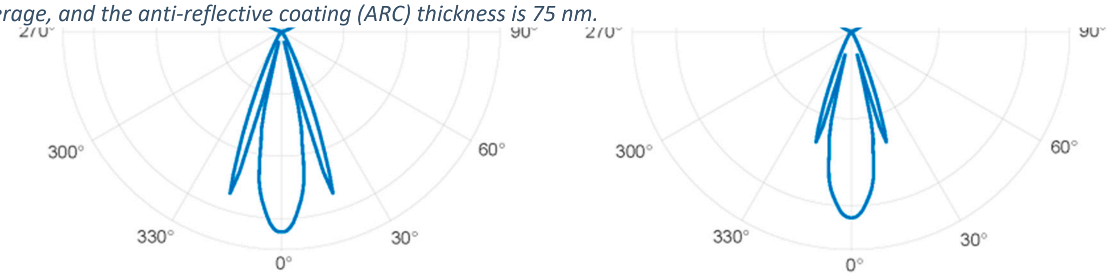

Figure S1: The angular scattering distribution at wavelength 613 nm from an Al nanoparticle with diameter 140 nm with a (a) 14 nm and (b) 42 nm oxide layer located on top of a c-Si substrate with a 75 nm ARC layer. The surface coverage is 21.0%.

Figure S2 shows the angular scattering distribution of the electric field intensity $|\mathrm{E}|^2$ for an Al nanoparticle with (a) a 14 nm and (b) a 42 nm thick oxide layer on top of a solar cell at wavelength 613 nm, where the ARC is most efficient. These are the oxide layer thicknesses yielding the smallest and largest increase in transmission for a 140 nm nanoparticle (see Fig. 4). Clearly, the side lobes in the forward direction are larger for a 14 nm oxide layer than for a 42 nm oxide layer, consequently reducing the efficiency of the ARC most in the former case (see discussion in Section 4.2). In addition, the maximum forward to maximum backward ratio is 3.1 for a 14 nm oxide layer and 5.2 for a 42 nm oxide layer. Hence, a larger portion of the incoming light is reflected for the nanoparticles with a 14 nm oxide layer than for the ones with a 42 nm oxide layer, further explaining the difference in total transmission for the two cases.

Figure S3 shows the normalized number of photons as a function of surface coverage for Al nanoparticles with a diameter of 140 nm and oxide layer of 42 nm on top of c-Si with a 75 nm ARC. The surface coverage yielding the largest increase in transmission is 21.0%.

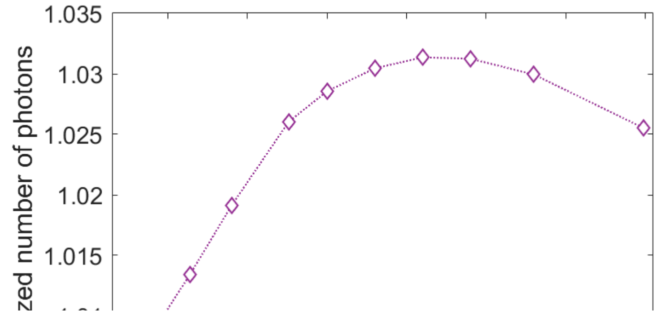

Figure S2: The normalized number of photons transmitted into c-Si as a function of surface coverage for Al nanoparticles with diameter 140 nm and a 42 nm oxide layer and a 75 nm thick ARC.

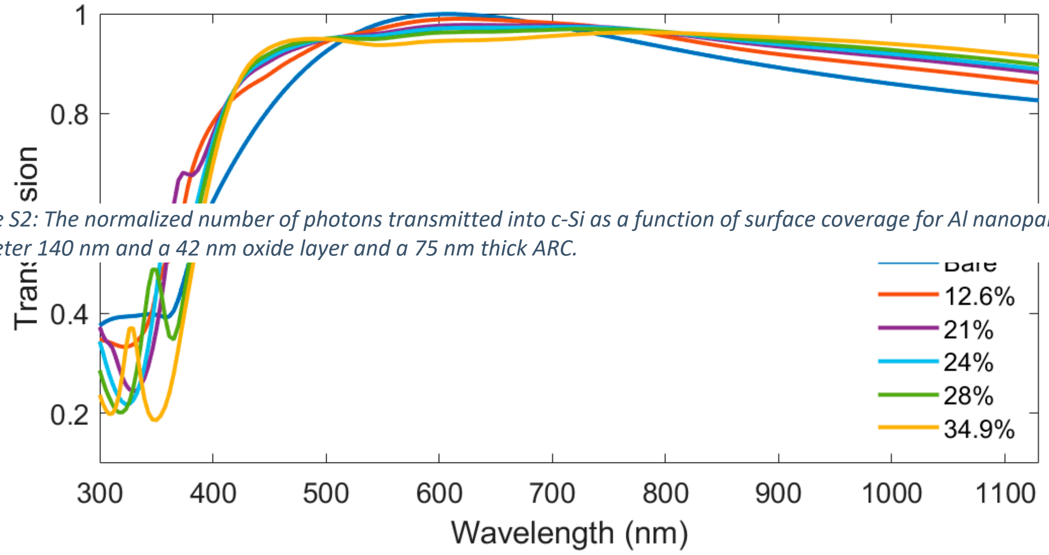

Figure S4 shows the transmission of light into c-Si as a function of wavelength for a bare solar cell and for a solar cell with Al nanoparticles with diameter 140 nm and oxide thickness 42 nm with varying surface coverage. The ARC thickness is 75 nm. The general trend for all surface coverages is the same as in Fig. S1. However, the Wood-Rayleigh anomalies are only visible for the 12.6% surface coverage, as the period is too small for it to be visible for higher surface coverages within the wavelength range used. We see that the peak below 400 nm caused by the LSPR of the nanoparticles is blue shifted with increasing surface coverage, as expected [53, 54]. Also, the amplitude of the peak increases due to the increasing number of nanoparticles.

Figure S3: The transmission of light into c-Si as a function of wavelength for a bare solar cell and for a solar cell with Al nanoparticles with varying surface coverage. The diameter of the nanoparticles is 140 nm with a 42 nm oxide layer and the ARC is 75 nm thick.

Figure S5 shows the transmission of light into c-Si as a function of wavelength for a bare solar cell and for a solar cell with Al nanoparticles with diameter 140 nm, oxide thickness 42 nm and surface coverage 21.0%. The ARC thickness varies from 60 nm to 100 nm. As expected, the wavelength yielding the highest transmission red-shifts as the ARC thickness increases. Figure S6 shows the transmission of light into c-Si for the same system using our EMT model. Clearly, the increase in transmission for short

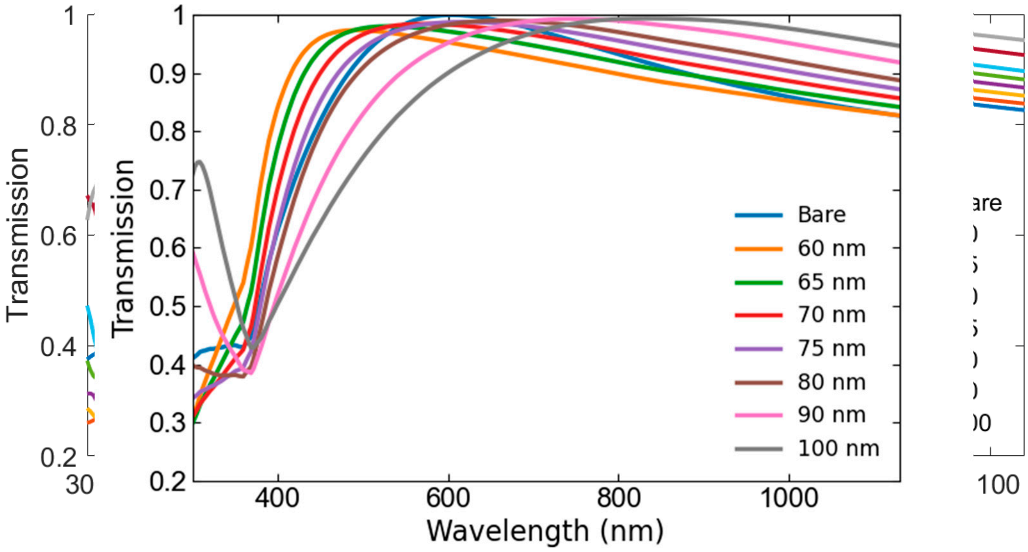

Figure S6: The transmission of light into c-Si for various ARC thicknesses as a function of wavelength for a solar cell with Al nanoparticles using the Fresnel equations with EMT.

wavelengths as the ARC thickness increases is caused by thin-film interference effects. The discrepancy in magnitude between the EMT plots and the simulated plots is caused by absorption in the nanoparticles and c-Si (see Section 4.2), and possibly loss due to interference between the scattered light from the nanoparticles and the reflected light from the substrate, which is not accounted for in the EMT model.

Figure S7 shows the angular scattered light distribution at a wavelength of (a) 640 nm (as in Fig. 8c) and (b) 390 nm for an angle of incidence of 54.7°. Clearly, the distribution is no longer symmetrical, as for normal incident light. At 640 nm, most of the back-scattered light will be scattered in the direction opposite the incident light. At 390 nm where the LSPR is, however, only slightly more of the back-scattered light will be scattered in the direction opposite the incident light. The scale in (a) is the same as in Fig. 8c for easy comparison.

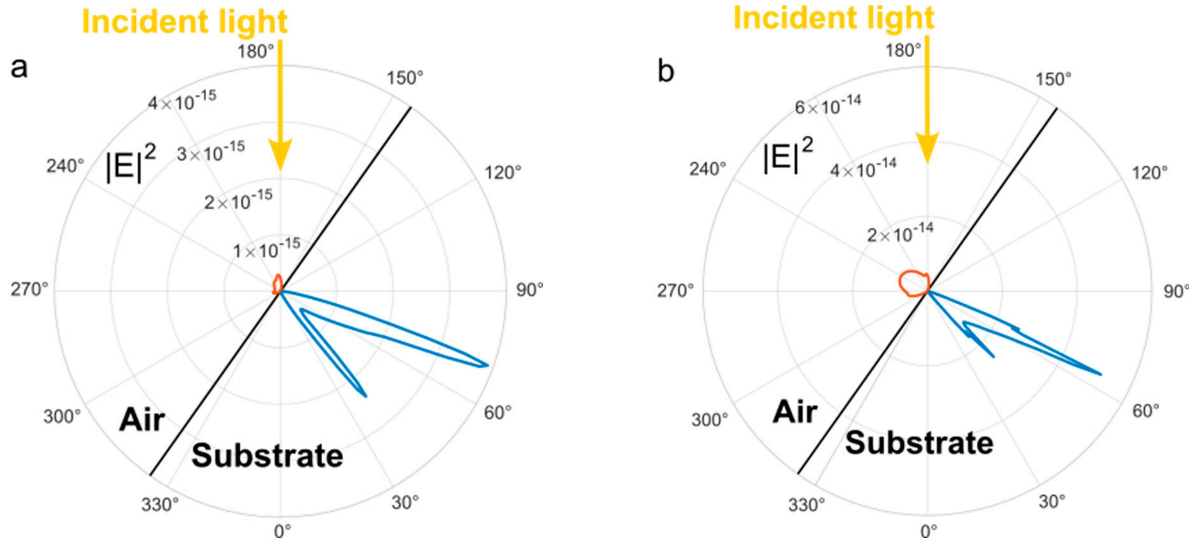

Figure S7: The angular scattering distribution for the scattered light at a wavelength of (a) 640 nm and (b) 390 nm for an incident light angle of 54.7°. Not that the scales are different, and that the scattering is an order of magnitude stronger at 390 nm, close to the LSPR of the nanoparticles.

S 2. Increase in Solar Cell Efficiency

The highest theoretical efficiency, η_{max}, that can be achieved with a single junction solar cell is determined by the Shockley-Queisser limit [2]. This limit can be divided into three factors: 1) The maximum collection efficiency, $\eta_{collection}^{max}$, 2) the bound on the amount of energy that can be extracted from the solar cell due to electron-hole recombination, eV_{oc}/E_{gap}, and 3) the fill factor, ff [55,56]:

$$\eta_{max} = \eta_{collection}^{max} \times \frac{eV_{oc}}{E_{gap}} \times \text{ff} \tag{S1}$$

When a solar cell is illuminated, only photons with energy greater than the band gap energy (E_{gap}) of the absorbing layer can excite electrons from the valence band to the conduction band, and the energy of less energetic photons is lost. Also, any energy beyond E_{gap} is lost to thermalization of the electrons in the conduction band. In total, this gives a limit on the collection efficiency of the solar cell. For silicon (Si), the maximum collection efficiency is approximately 49% [55]. Furthermore, there is an upper bound on the open circuit voltage V_{oc} due to electron-hole recombination. The energy absorbed from each photon by the solar cell is E_{gap}, but only eV_{oc} of this energy can be extracted, and the factor eV_{oc}/E_{gap} limits the total efficiency of the solar cell. The upper bound on this factor is approximately 0.76 for a Si solar cell [57]. The fill factor is the ratio of the maximum total power to the product $I_{sc}V_{oc}$, where I_{sc} is the short circuit current. The theoretical maximum value of the fill factor is approximately 0.87 for Si [57]. In total, the maximum theoretical efficiency of a Si solar cell is approximately 32%.

The last two terms in Eq. S1 depend solely on the material properties of the solar cell and is not affected by the addition of nanoparticles on the top surface of the solar cell. On the contrary, the first term also depends on the energy and intensity of the incident light. To make a simple estimate on the increase in overall efficiency of a Si solar cell when adding nanoparticles, we assume the solar cell has optimized material properties. The overall efficiency is then 32% when an energy E_{gap} is collected from all incident photons with energy $E \geq E_{gap}$. Eq. S1 can then be used to calculate the overall efficiency of the solar cell. The last two terms can be held constant as $\frac{eV_{oc}}{E_{gap}} \times \text{ff} = 0.76 \times 0.87 \approx 0.66$. The overall efficiency is then

$$\text{Overall efficiency } = 0.66 \times \eta_{collection} \quad \text{(S2)}$$

$\eta_{collection}$ is the ratio of the energy absorbed by the solar cell to the total energy incident on the solar cell. The total energy hitting 1 m^2 of the solar cell is, using the AM1.5: ASTM G-173-03 solar spectrum [40], $6.2444\text{e}24 \text{ J/s}/\text{m}^2$. The energy absorbed by the solar cell is found by first calculating the number of photons transmitted into the solar cell in the wavelength range 300 to 1130 nm, and then assume that 1.1 eV is absorbed from each photon. This is done for a bare solar cell and for a solar cell with nanoparticles with optimized parameters on top. The transmitted energy for a bare solar cell is $2.7447\text{e}21 \text{ J/s}/\text{m}^2$, yielding an overall efficiency of 29.06%. The transmitted energy for a solar cell with nanoparticles is $2.8360 \text{ J/s}/\text{m}^2$, yielding an overall efficiency of 30.03%. The absolute increase in solar cell efficiency when nanoparticles are introduced is then $0.966\% \approx 1.0\%$.

References

[53] B. Lamprecht, G. Schider, and R. T. Lechner, et al., “Metal Nanoparticle Gratings: Influence of Dipolar Particle Interaction on the Plasmon Resonance,” Phys. Rev. Lett., vol. 84, pp. 4721–4724, May 2000, doi: 10.1103/PhysRevLett.84.4721.

[54] C. L. Haynes, A. D. McFarland, and L. Zhao, et al., “Nanoparticle Optics: The Importance of Radiative Dipole Coupling in Two-Dimensional Nanoparticle Arrays,” J. Phys. Chem. B, vol. 107, no. 30, pp. 7337–7342, 2003, doi: 10.1021/jp034234r.

[55] R. L. Jaffe and W. Taylor. *The Physics of Energy*. Cambridge: Cambridge University Press, 2018. doi: 10.1017/9781139061292

[56] P. T. Landsberg and T. Markvart, “Chapter IA-3 - Ideal Efficiencies,” in: Editor(s): A. McEvoy, L. Castañer, T. Markvart (eds.), *Solar Cells,* 2nd ed., Amsterdam, Netherlands, Elsevier, 2013, pp. 55-66. https://doi.org/10.1016/B978-0-12-386964-7.00003-2

[57] S. Rühle, Tabulated values of the Shockley–Queisser limit for single junction solar cells, *Solar Energy*, vol. 130, pp. 139-147, 2016. https://doi.org/10.1016/j.solener.2016.02.015
